# Expression and characterization of novel ovine orthologs of bovine placental prolactin-related proteins

**DOI:** 10.1186/1471-2199-8-95

**Published:** 2007-10-25

**Authors:** Koichi Ushizawa, Toru Takahashi, Misa Hosoe, Katsuhiro Ohkoshi, Kazuyoshi Hashizume

**Affiliations:** 1 Reproductive Biology Research Unit, Division of Animal Sciences, National Institute of Agrobiological Sciences, 2 Ikenodai, Tsukuba, Ibaraki 305-8602, Japan; 2 Department of Veterinary Medicine, Faculty of Agriculture, Iwate University, 3-18-8 Ueda, Morioka, Iwate 020-8550, Japan

## Abstract

**Background:**

The prolactin-related proteins (PRPs) are non-classical placental-specific members of the  prolactin/growth hormone family. Among ruminants, they are expressed in the cotyledonary villi of cattle and goat. We investigated placental PRP in sheep in order to gain a comprehensive understanding of the function and evolution of these molecules. We also examined the sequence properties, expression and lactogenic activation of the cloned genes.

**Results:**

We cloned two novel ovine *PRPs*, named *oPRP1 *and *oPRP2*. *oPRP2 *had a typical *PRP *sequence similar to bovine *PRP1 *(*bPRP1*). *oPRP1 *had a short sequence identical with bovine or caprine type *PRP *but the reading frame was shifted. Both *oPRPs *were expressed in trophoblast giant binucleate cells (BNC) as in cattle and goat. *oPRP1 *expression declined from the early to the middle stage of gestation. In contrast, *oPRP2 *expression remained constant throughout the gestation period. *oPRP2 *was translated to form a mature protein in a mammalian cell expression system. Western blotting showed a molecular mass of 35 kDa for the FLAG-tag fusion *oPRP2* protein. This recombinant protein and *bPRP1* were bioassayed using Nb2 lymphoma cells; it was confirmed that neither ruminant *PRP* had lactogenic activity because the Nb2 lymphoma cells did not proliferate.

**Conclusion:**

We have identified two novel *PRPs*, *oPRP1 *and *oPRP2*, in ovine placenta. Both these ovine *PRPs* were localized and quantitatively expressed in BNC. Absence of lactogenic activity was confirmed for the *oPRP2* molecule. It is anticipated that novel and known ruminant *PRPs* have common functions, except for lactogenic activity.

## Background

Prolactin-related proteins (PRPs) are non-classical members of the prolactin (PRL)/growth hormone (GH) family that have been found in bovine, caprine, murine and rat placenta. In cattle, placental lactogen (PL) and thirteen types of placental PRPs have so far been reported [1-3]. In goats, PL has been detected in the placenta [4], and two newly-discovered PRPs were reported as PRL-related molecules in our recent study [5]. In ruminants, specifically in cattle, sheep and goats, trophoblast-specific genes such as *PL *[4,6,7], pregnancy-associated glycoproteins (PAGs) [8-11], interferon-tau (*IFNT*) [12-14], trophoblast kunitz domain proteins (TKDPs) [15,16] and cathepsins (CTSs) [17,18] are known to be key factors for implantation and placentation, and are expressed in trophoblast cells including trophoblast giant binucleate cells (BNC). However, no molecules similar in sequence to bovine and/or caprine PRPs have been reported in sheep. It is assumed that PRP orthologs are involved in ovine placenta because placental PRL-like molecules have been discovered in many mammals, not only in cattle and goat but also in mouse and rat [1-3,19]. In the present study, we identified the mRNAs of two novel PRPs in ovine placenta and investigated their expression in the ovine placentome. We named them ovine prolactin-related protein-1 (*oPRP1*) and prolactin-related protein-2 (*oPRP2*) on the basis of similarities with cattle and goat sequences. They were translated in a HEK293 cell transfection system, as in the case of cattle [20,21]. Their lactogenic activities were confirmed by an Nb2 lymphoma cell bioassay [22-24]. An aim of future research will be to determine the function of ruminant PRP molecules that appear in the placenta. A crucial part of such a study will be to express ruminant PRP in other species. The purposes of the present study are (i) to explore a bPRP homolog gene in ovine placenta, (ii) to investigate the localized and quantitative expression of oPRP and (iii) to examine the possible biological activity of oPRP, because comparison among ruminant PRP structures and/or expression may provide clues to understanding PRP function.

## Results

### oPRP1 and oPRP2 nucleotide sequences and deduced amino acid sequences

**Figure 1:**
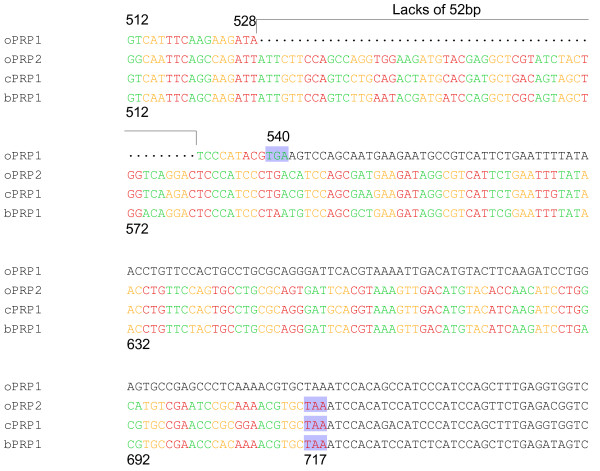
**The stop codon region of *PRPs *mRNA**. *oPRP2*, *bPRP1 *and *cPRP1 *have a stop codon 717 bp from the CDS start site. In oPRP1 the stop codon is shifted to 540 bp from the CDS start. The shaded boxes indicate the stop codon. The sequence gaps are shown by dots.

Full-length *oPRP1 and oPRP2 *were cloned from the ovine placentome on day 95 of gestation. *oPRP1 *was 893 nucleotides (nts) long with a 540-nts protein coding sequence region (CDS); *oPRP2 *had a 947-nts full-length sequence and a 717-nts CDS. The protein sequences deduced from the full-length cDNAs comprised 179 amino acids (aa) in oPRP1 and 238 aa in oPRP2. The sequence region in which *oPRP1 *mRNA is defective compared to other PRP mRNAs is shown in Fig. 1 along with the sequences of *oPRP2*, *cPRP1 *and *bPRP1*. *oPRP1 *has a shorter sequence, lacking 52 bp from the CDS regions of other *PRP *sequences (positions 529–580). Since the reading frame of the codon is shifted at the 52 bp defect, the CDS region became 540 bp.

**Figure 2:**
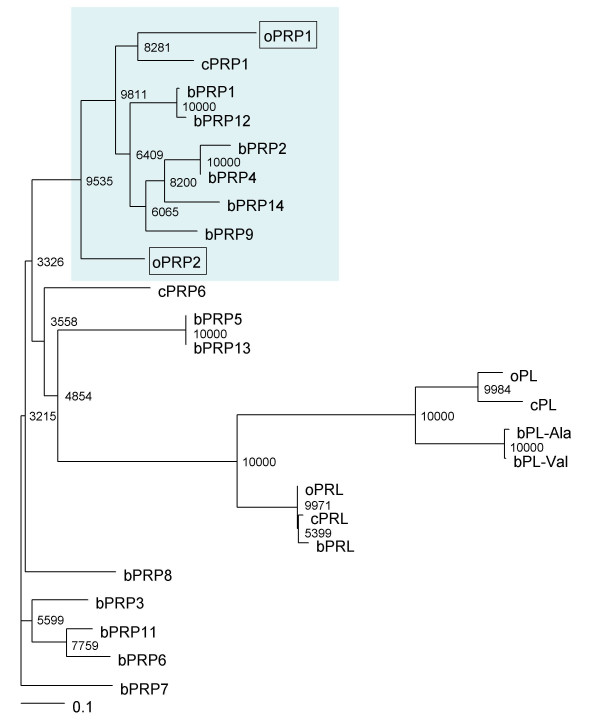
**Phylogenetic tree of prolactin and placental-prolactin family of sheep, cattle and goat**. The tree was constructed using TreeView following the alignment of protein sequences given by the Clustal W 1.83 algorithm. The numbers at the base of each branch division represent bootstrap values after 10,000 repeats. The scale bar represents 0.1 amino acid replacements per amino acid site. For GenBank/DDBJ accession numbers, refer to Materials and Methods. The proteins in light blue areas were used for the multiple alignments in Fig. 3.

Fig. 2 shows a phylogenetic tree analysis based on the predicted aa sequences of the new oPRPs and other prolactin family members in cattle and goat. We confirmed a close phylogenetic relationship between oPRP1 and bPRP1, bPRP2, bPRP4, bPRP9, bPRP12, bPRP14 and cPRP1, which have known sequences. oPRP2 was considerably more distant from bPRP1, bPRP2, bPRP4, bPRP9, bPRP12, bPRP14 and cPRP1 in the phylogenetic tree analysis.

**Figure 3:**
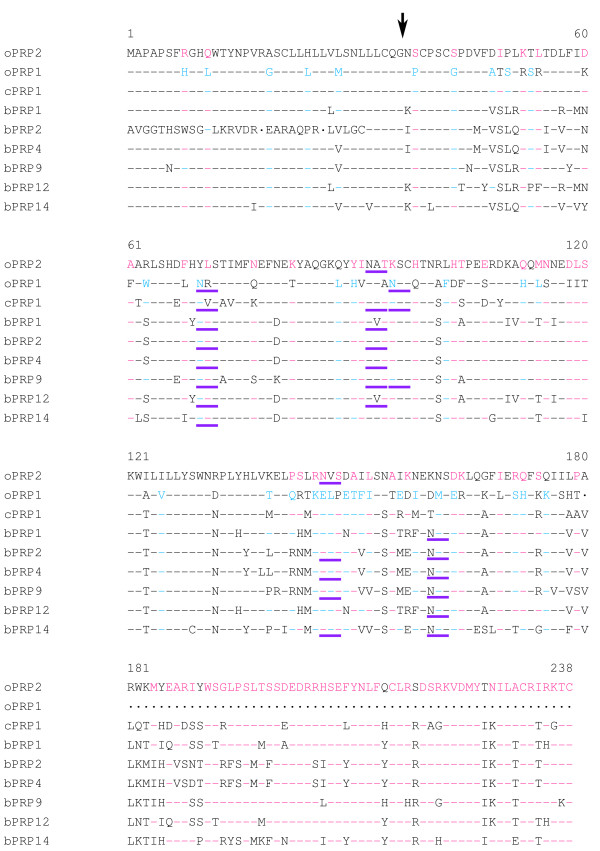
**Comparison of amino acid sequences of oPRP1 and oPRP2 with phylogenetically neighbouring PRPs**. Residues identical to oPRP1 and oPRP2 are shown by black hyphens, residues present only in oPRP2 by pink hyphens, and residues present only in oPRP1 by blue hyphens. The sequence gaps are shown by dots. The amino acid sequences were aligned with the help of Clustal W 1.83 on the DDBJ web site. The arrow indicates the putative primary cleavage site of the signal peptide of oPRP1 or oPRP2. The potential *N*-glycosylation site is underlined in purple.

The identity of oPRP1 and oPRP2 with the phylogenetically neighbouring PRPs is demonstrated in Fig. 3 and Table 1. The *N*-terminal regions of the oPRP1 and oPRP2 proteins are rich in hydrophobic amino acid residues, which is characteristic of signal peptides. The signal peptide sequence, which is composed of 36 amino acids, is well conserved in the bPRP and/or cPRP family [1,5]. The mature oPRP1 protein is predicted to have one disulfide bond with three cysteines (Cys). In contrast, the mature oPRP2 protein is predicted to have three disulfide bonds with six Cys. Normally, Cys is common to the 39, 42, 97, 215, 232 and 238 positions in bPRP1, bPRP2, bPRP4, bPRP9, bPRP12, bPRP14 and cPRP1 (Fig. 3). In contrast, oPRP1 lacked the Cys at positions 215, 232 and 238, because there is no sequence corresponding to positions 180–238 (Fig. 3). oPRP1 has one potential consensus sequence for typical *N*-glycosylation, an Asn-X-Ser/Thr (NXS or NXT) at positions 70–72 (Fig. 3). oPRP2 has two consensus sequences for *N*-glycosylation at positions 92–94 and 146–148 (Fig. 3). Another atypical *N*-glycosylation site, Asn-X-Cys (NXC), was exhibited at positions 95–97 in oPRP1 (Fig. 3). The two positions in oPRP2 coincided with those in bPRP2, bPRP4, bPRP9 and bPRP14 (Fig. 3) [1,20,21]. In contrast, the typical position (70–72) in oPRP1 coincided with those in bPRP1 and cPRP1, while the atypical position (95–97) coincided with those in cPRP1 and bPRP9 [1,5,20,21].

**Table 1:** Identity of oPRP1 and oPRP2 for phylogenetically neighbouring PRPs (%)

	bPRP1	bPRP2	bPRP4	bPRP9	bPRP12	bPRP14	cPRP1
oPRP1	64	53	66	67	63	63	73
oPRP2	71	60	71	71	71	68	71

**Figure 4:**
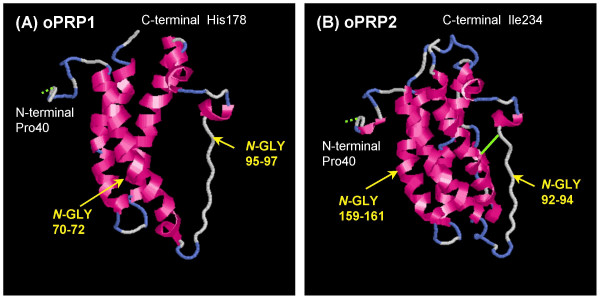
**The predicted 3D structures of mature (A) oPRP1 and (B) oPRP2 proteins**. The 3D structures were predicted by FAMS software. The oPRP1 structure was constructed in the Pro40-His178 region. The oPRP2 structure was constructed in the Pro40-Ile234 region. Disulfide bonds are shown as light green solid lines, predicted disulfide bonds as light green dotted lines. *N*-GLY indicates the potential *N*-glycosylation site.

The predicted 3D structures of mature oPRP1 and oPRP2 are illustrated in Fig. 4. In general, PRL family members are predicted to have four α-helices, like oPRP2, but oPRP1 may have only three α-helices (Fig. 4). The *oPRP1 *sequence has a premature stop codon because the reading frame is shifted; residues 529–580 (52 bp) in the other PRP sequences are absent (Fig. 1). The deduced molecular structure predicts that this protein lacks the fourth α-helix found in existing bovine or caprine PRPs (Fig. 4). Structural differences in the *N*-glycosylation site, the disulfide bond (-S-S-) between Cys97 and Cys215 and each atomic configuration were also exhibited (Fig. 4).

The *oPRP1 *and *oPRP2 *sequences were submitted to the DNA Data Bank of Japan (DDBJ); the DDBJ/GenBank accession numbers are AB231297 and AB231298.

### Localization and quantitative expression of *oPRP1 *and *oPRP2 *mRNA

**Figure 5:**
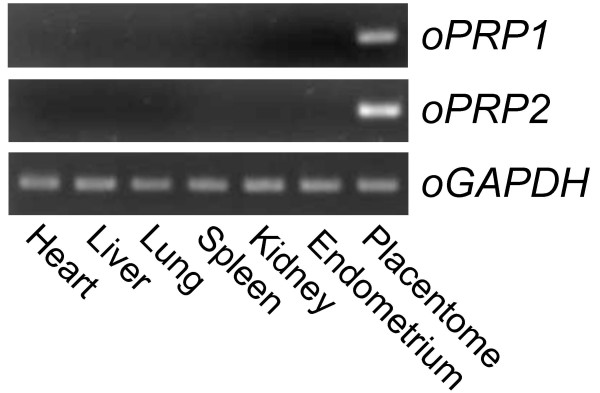
**Expression of *oPRP1 and oPRP2 *mRNA in ovine tissues**. Heart, liver, lung, spleen, kidney and endometrium were used for RT-PCR. Cotyledonary tissue at Day 45 of gestation was used as a placental sample. *GAPDH *expression in each tissue is presented as a control.

Specific expression of *oPRP1 *and *oPRP2 *mRNA was detected in ovine placenta (Fig. 5) by conventional RT-PCR. No signal was observed in other ovine tissues, i.e. heart, liver, lung, kidney, spleen and endometrium.

**Figure 6:**
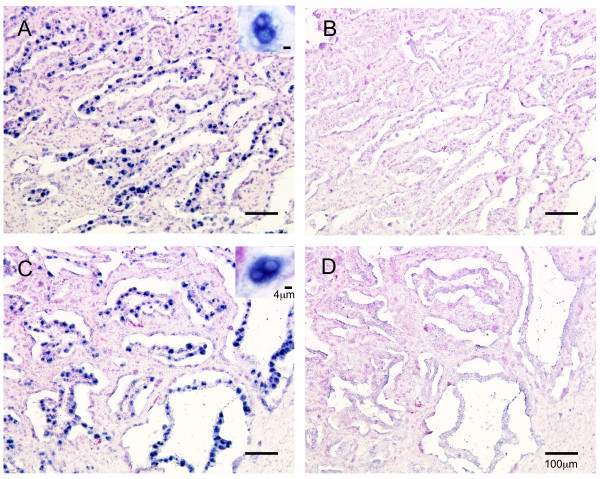
**Localization of *oPRP1 *and *oPRP2 *in ovine placentome on Day 45 of gestation**. (A, B) *oPRP1 *and (C, D) *oPRP2 *mRNAs were detected by *in situ *hybridization. (A, C) DIG-labeled anti-sense cRNA probes were used. (B, D) DIG-labeled sense cRNA probes were used. Seven micrometer sections of ovine placentome were hybridized with each probe. Scale bars = 100 μm (main areas in A, B, C and D) and 4 μm (right upper areas in A and C).

*oPRP1 *and *oPRP2 *mRNAs were localized by *in situ *hybridization in the ovine placentome (Fig. 6). DIG-labeled *oPRP1 *and *oPRP2 *anti-sense RNA probes specifically detected the mRNA transcript in the placentomes. Both *oPRP1 *and *oPRP2 *appeared in the BNC in the cotyledonary villi area (Fig. 6). No significant *oPRP1 *or *oPRP2 *signals were detected using sense probes (Fig. 6).

**Figure 7:**
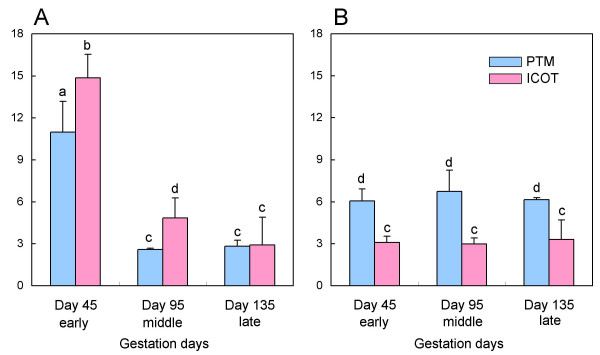
**QPCR analysis of (A) *oPRP1 *and (B) *oPRP2 *mRNA in ovine placenta**. Total sheep RNA was extracted from PTM and ICOT on Day 45 (early), Day 95 (middle) and Day 135 (late) of gestation. Expression of these mRNAs was normalized to the expression of *GAPDH *measured in the corresponding RNA preparation. Values are means ± SEM. Values with different letters (a, b, c and d) are significantly different (*P *< 0.05).

Quantitative expression of *oPRP1 *and *oPRP2 *is shown in Fig. 7. In the placentomal tissue (the cotyledonary and caruncular parts: PTM), the intensity of *oPRP1 *expression declined from Day 45 to Day 95 and then remained constant to Day 135. The intensity of *oPRP2 *expression remained constant between the early (Day 45) and late (Day 135) stages of gestation. In the intercotyledon (the membrane between the cotyledonary villi: ICOT), *oPRP1 *expression declined from the early (Day 45) to the late (Day 135) stages of gestation. Again, as in the placentome, *oPRP2 *expression remained constant between the early (Day 45) and late (Day 135) stages. There was more intense *oPRP1 *expression in ICOT than in PTM during the early (Day 45) to middle (Day 95) stages of gestation. In contrast, *oPRP2 *expression was greater in the PTM than ICOT throughout pregnancy.

### Production of recombinant proteins

**Figure 8:**
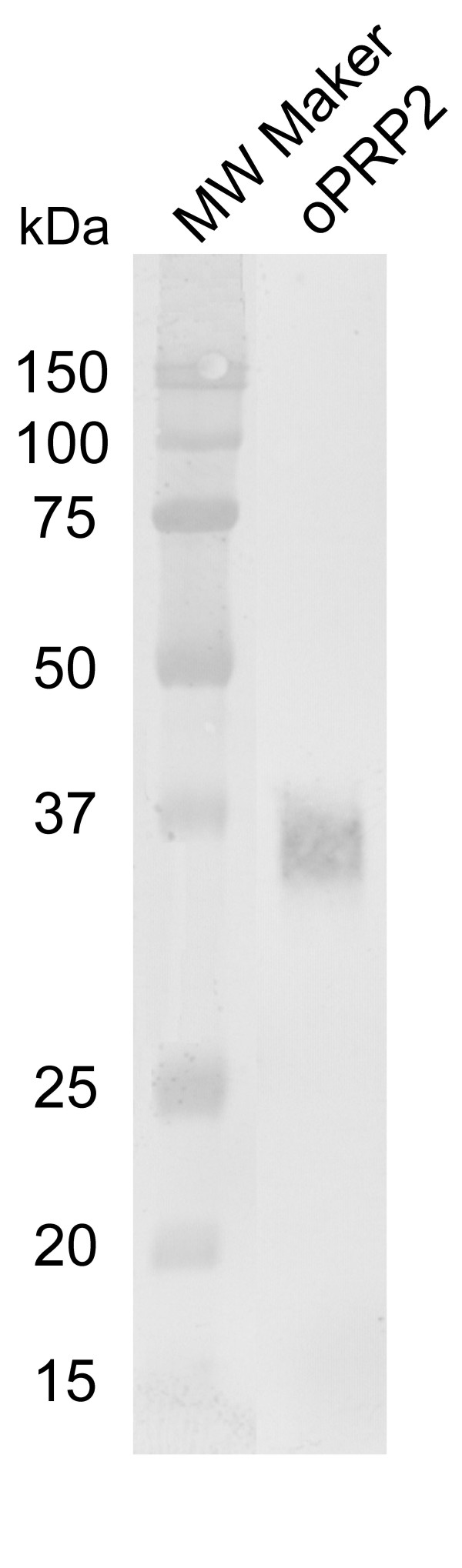
**Western blot analysis of recombinant FLAG-tag fusion oPRP2 protein**. Conditioned media from HEK 293 cells transiently transfected with each gene were collected, and the purified proteins (1 ng) were loaded on to separate lanes. The proteins were separated by SDS-PAGE and specific proteins were detected by Western blot analysis using an anti-FLAG antibody. MW Marker: molecular weight marker.

We produced oPRP2 recombinant protein in order to investigate its lactogenic activity. Cloned *oPRP2 *sequences were efficiently translated in an HEK293 cell system, as in the case of bovine PRPs (Fig. 8) [20,21]. A FLAG-tag fusion oPRP2 protein was translated at approximately 35 kDa (Fig. 8). We also tried to produce recombinant oPRP1 protein in a mammalian cell system but were unable to do so.

### Lactogenic activity of PRP

**Figure 9:**
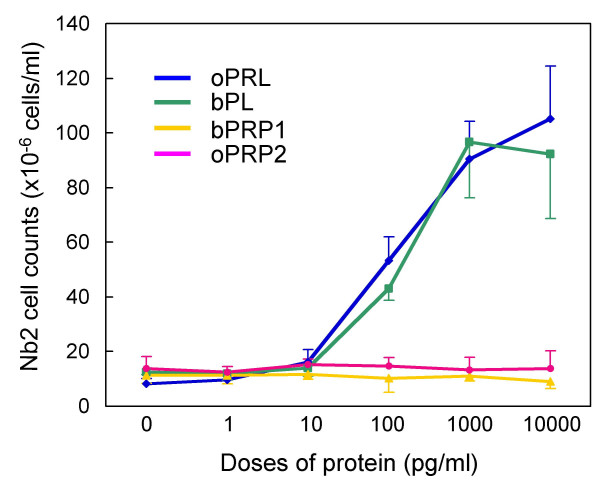
**Lactogenic activity of oPRP2 and bPRP1**. Nb2 lymphoma cell proliferation and PRP dosage are shown. oPRL and bPL were used as positive controls. Values are means ± SD.

Ovine PRL (positive control) stimulated Nb2 lymphoma cell proliferation effectively, and another lactogenic protein, bovine PL, showed stimulatory activity (Fig 9). No stimulatory activity was detected in recombinant oPRP2, as for bPRP1.

## Discussion

The diversity of the PRL gene family has been demonstrated in mouse, rat, cattle and goat, but functional information about these proteins is limited except for PRL, PL and some rodent prolactin-like proteins (Prlps) [2,19,25-29]. In particular, ruminants commonly have various genes of this family, but there is no information regarding sheep, even though anatomical evidence shows that placentae are similar among bovidae [1,20,21,30,31]. Thirteen varieties of PRP paralogs have been reported in bovines and two in goat [1,5,20,21]. In the present study, we have identified novel PRPs in sheep and compared the properties of sheep and cattle/goat.

The novel *oPRP1 *and *oPRP2 *genes were detected in and cloned from ovine placenta and oPRP1 was deduced to have a shortened amino acid sequence. Phylogenetic analysis suggests that PRP molecules evolved as ruminant species diverged, because these ovine PRPs are phylogenetically adjacent to caprine and bovine PRPs (Fig. 2).

We confirmed the short variant form of PRP in ovine placenta (Figs. 3 and 4). Although there is no report that short variant forms result from alternative splicing or proteolytic cleavage in non-classical members of the PRL family, namely bovine PRP, caprine PRP, mouse Prlp and rat Prlp [1,2,5,25,26], short variants of bPL and PRL have been reported [32,33]. Two types of short *bPL *variant are formed by alternative splicing [34]. One has a premature stop codon because of a shift in the reading frame. Although the defective position in the *bPL *variant sequence was similar to that in *oPRP1*, *bPL *lacked only 23 bp in this region. It is not yet known whether the short bPL variant exists or what function it may have. Some structural variants of PRL proteins have been confirmed in various mammalian species [32]. A short fragment of PRL protein has also been confirmed in rat, mouse and human [35-37]. Although these short PRL (16 kDa-PRL) molecules may result from alternative splicing, they could also be generated by proteolytic cleavage. The cleavage site (positions 145–149 in the mature region) almost coincides with the position of the *oPRP1 *stop codon. These short PRLs in mouse and human are known to have potential as inhibitors of capillary endothelial cell proliferation [38,39]. The short PRL in rat produces an anti-angiogenic effect via an unique, high-affinity, saturable receptor that is different from the PRL receptor [40]. Whether oPRP1 corresponds to the short PRL receptor is not certain, because full-length PRL binds to the PRL receptor and some ruminant PRPs do not. However, one hypothesis may be that oPRP1 has an inhibitory effect on the proliferation of vascular endothelial cells, because the sequence length and the mature protein region are similar to those of the short PRL variants in human and rodents. Members of the rodent Prl superfamily, namely prolactin-like proteins (Prlp), proliferins (Plf) and proliferin-related protein (Plfr), are non-classical and have several specific activities such as angiogenesis [41-43], hematopoiesis [44-46] and immunomodulation [47-50]. A possible hypothetical function for oPRP1 may be in the regulation of angiogenesis, but its actual function remains to be clarified.

Primary expression of *oPRP1 *and *oPRP2 *mRNA was observed in BNC (Fig. 6). The localization of *oPRP *mRNAs is similar to that of PRP family members in other ruminants [5,20,21,30,31,51]. The mRNAs of both oPRPs were detected in the PTM and ICOT tissues throughout pregnancy (Fig. 7). However, the expression patterns were different; *oPRP1 *expression declined with the progress of the pregnancy in PTM and ICOT, but in contrast, *oPRP2 *expression remained constant throughout pregnancy. However, there seems to be a discrepancy between the localization and quantitative expression of *oPRP2*: quantitative real-time RT-PCR (QPCR) data suggest that *oPRP2 *may be expressed not only in BNC but also in conventional trophoblast cells, as in bovine [20,21]. In *bPRP *expression profiles, four types of expression pattern were found during pregnancy in PTM: (i) genes expressed around the window; (ii) genes with peak expression around mid-gestation; (iii) genes that show increasing expression during the progression of gestation and peak late in gestation; (iv) genes with approximately constant expression throughout gestation [34]. In the present study, although expression was not determined during the implantation period, *oPRP1 *expression may correspond to the type (ii) pattern. In contrast, *oPRP2 *expression might be type (iv) but without lactogenic activity. The functional significance of short PRPs and various other kinds of PRP in ruminant placenta is still unclear. We could produce recombinant oPRP2 protein as well as proteins of other ruminant species (cattle and goat) [5,20,21]. Only the absence of lactogenic activity is clearly confirmed for recombinant oPRP2 and bPRP1 by a bioassay using Nb2 lymphoma cells.

## Conclusion

We have cloned two novel prolactin-related protein genes in ovine placentome. The ovine *PRP *sequences have a high homology with bovine *PRP*. However, *oPRP1 *has a premature stop codon, which has not been discovered in bovine and caprine *PRPs*. *oPRPs *were expressed in trophoblast BNC, as are bovine or caprine *PRPs*. Their mRNAs were expressed throughout gestation. *oPRP1 *mRNA declined with the progress of gestation; *oPRP2 *mRNA remained constant throughout. *oPRP2 *produced mature recombinant protein in a mammalian cell-expression system. We confirmed that oPRP2 is lactogenically inactive, as oPRP2 treatment did not induce proliferation of Nb2 lymphoma cells.

## Methods

### Animals and tissues collection

Ovine placental tissues for cDNA cloning, mRNA expression and *in situ *hybridization were collected from Corriedale sheep. The PTM and ICOT were collected at a local slaughterhouse on Days 45 (n = 3 animals), 95 (n = 3 animals) and 135 (n = 2 animals) of gestation after natural mating (day 1). The collected samples were stored at -80°C prior to RNA extraction. The placentomes were fixed in 3.7% formaldehyde PBS at pH 7.4 and then embedded in paraffin wax and stored at 4°C prior to *in situ *hybridization. 

All procedures for these animal experiments were carried out in accordance with the guidelines and ethics approved by the Animal Ethics Committee of the National Institute of Agrobiological Sciences for the use of animals.

### Cloning of full-length *oPRP *cDNAs

Full-length cDNAs of the novel *oPRP1 and oPRP2 *were isolated from ovine cotyledonary tissue by the 3'-rapid amplification of cDNA ends (RACE) method. In brief, complete RNA was isolated from an ovine placentome on day 45 of gestation using ISOGEN (Nippon Gene, Toyama, Japan). We performed 3'-RACE using a 3'-full RACE core set (Takara, Kyoto, Japan) with an *oPRP*-specific forward primer (5'-CTATGGTCAACAGGCGTCCTCA-3'). The *oPRP *primers were designed from bovine *PRP *sequences. The 3'-RACE products were sequenced using an ABI Prism 370 automatic sequencer (Applied Biosystems, Foster City, CA, USA) after cloning into a pGEM-T Easy vector (Promega, Madison, WI, USA).

### Phylogenetic analysis

The deduced oPRP1 and oPRP2 protein sequences were aligned with the ruminant PRPs using the multiple alignment software Clustal W 1.83 on the DDBJ web site. Clustal W was also employed to calculate trees using the Neighbor-Joining (NJ) method [52]. TreeView was used to display the phylogenetic tree [53]. The values represent bootstrap scores for 10,000 trials, indicating the credibility of each branch. Except for the oPRP1 and oPRP2 sequences, the ruminant PRL family protein sequences were obtained from GenBank. Their GenBank accession numbers are: bPRP1 (J02944), bPRP2 (M27239), bPRP3 (M27240), bPRP4 (M33269), bPRP5 (X15975), bPRP6 (AB245482), bPRP7 (AB187564), bPRP8 (AB196438), bPRP9 (AB204881), bPRP11 (BK005438), bPRP12 (BK005439), bPRP13 (BK005440), bPRP14 (AB255602), bPL-Ala (J02840), bPL-Val (M33268), bPRL (V00112), oPL (M31660), oPRL (M27057), caprine PRL (cPRL: X76049), cPRP1 (AB231295) and cPRP6 (AB231296). The cPL sequence was obtained from Sakal et al. [4].

### Three-dimensional structure prediction

We predicted the three-dimensional (3D) structures of oPRP1 and oPRP2 using FAMS (Fully Automated Homology Modeling System) [54,55]. FAMS is a software program that predicts 3D models for target proteins on the basis of the structures of proteins that are known to be highly homologous. For oPRP1 and oPRP2, the 3D structure was constructed on the basis of that of human prolactin (hPRL) (Protein Data Bank ID: 1N9D). The FAMS program requires only an amino acid sequence as input and constructs 3D model structures automatically. The 3D structures were visualized using RasMol 2.7.3 software [56,57].

### RT-PCR

The tissue distribution of *oPRP1 *and *oPRP2 *expression was studied by RT-PCR. Ovine *GAPDH *was used as a positive control. Details of the RT-PCR method were described previously [20,21]. The total RNA in a reaction mixture was used for reverse transcription and template cDNA synthesis using oligo(dT) primers and Superscript III reverse transcriptase (Invitrogen, Carlsbad, CA, USA) at 50°C for 50 min. Each PCR contained the cDNA template, primers, deoxynucleotide triphosphate mixture (dNTP), MgCl_2_, 10 × PCR buffer II, autoclaved milliQ water and AmpliTaq gold DNA polymerase (Applied Biosystems). Amplification conditions included denaturation at 95°C for 30 s and extension at 72°C for 1 min. Twenty-seven cycles were performed for all samples. The annealing temperature was set at 58°C for 30 s. A single denaturation step at 95°C for 10 min before the first PCR cycle and a final extension step at 72°C for 10 min after the last PCR cycle were also performed. The PCR products were analyzed by agarose gel electrophoresis and visualized by ethidium bromide staining. The primers encoding the *oPRP1 *and *oPRP2 *sequences were designed using our obtained sequence. The designated primers are listed in Table 2. The primers were commercially synthesized (Tsukuba Oligo Service, Tsukuba, Japan).

**Table 2:** Oligonucleotide primers used for RT-PCR analysis

Gene	Primer	Sequence	Position
*oPRP1*	Forward	5' AACCCATGCCCGTCCTGCGGT 3'	157–177
(AB231297)	Reverse	5' TTAGCACGTTTTGAGGGCTCG 3'	714–694
*oPRP2*	Forward	5' AACTCATGCCCATCCTGCAGT 3'	155–175
(AB231298)	Reverse	5' TTAGCACGTTTTGCGGATTCG 3'	763–743
*oGAPDH*	Forward	5' AAGGCCATCACCATCTTCCA 3'	78–97
(AF030943)	Reverse	5' AGGTCAGATCCACAACGGACA 3'	603–583

### *In situ *hybridization

The full-length *oPRP1 *and *oPRP2 *cDNAs were used as templates for hybridization probe synthesis. Digoxigenin (DIG)-labeled antisense and sense-complementary RNA probes were prepared as described in previous studies [20,21]. The placentomes were sectioned into 7 μm-thick sections. *In situ *hybridization was performed using automated Ventana HX System Discovery with a RiboMapKit and BlueMapKit (Ventana, Tucson, AZ, USA) [20,21]. Briefly, ovine placentomal  sections were hybridized with DIG-labeled probes in RiboHybe (Ventana) hybridization solution at 67°C for 6 hours. The sections were washed three times in RiboWash (Ventana) (67°C, 6 min) after hybridization and fixed in RiboFix (Ventana) (both 37°C, 10 min). The hybridization signals were then detected using a monoclonal-anti-digoxin biotin conjugate (Sigma, Saint Louis, MI, USA). The hybridized slides were observed after preparation with a Leica DMRE HC microscope (Leica Microsystems, Wetzlar, Germany) with a Fujix digital camera HC2500 (Fujifilm, Tokyo, Japan).

### Quantitative real-time RT-PCR (QPCR)

Expression of *oPRP1 *and *oPRP2 *was confirmed quantitatively at each stage of gestation by QPCR using the Power SYBR Green PCR master mix (Applied Biosystems). Fifty ng of total RNA was reverse-transcribed into cDNA for 30 min at 48°C using MultiScribe™ reverse transcriptase with a random primer, dNTP mixture, MgCl_2 _and RNase inhibitor. After heat inactivation of the reverse transcriptase for 5 min at 95°C, PCR and the resulting relative increase in reporter fluorescent dye emission were monitored in real time using an Mx3000P QPCR system (Stratagene, La Jolla, CA, USA). In the SYBR Green assay, primer pairs were designed using the Primer Express software program (Applied Biosystems). The primers for each gene are listed in Table 3. Thermal-cycling conditions included initial-sample incubation at 50°C for 2 min and at 95°C for 10 min, followed by 40 cycles at 95°C for 15 s and at 60°C for 1 min. The relative differences in the initial amounts of each mRNA (or cDNA) species were determined by comparing the C_T _values. Standard curves for each gene were generated by serial dilution of the plasmid containing the corresponding cDNA to quantify the mRNA concentrations. We confirmed the melting curve for detecting the SYBR Green-based objective amplicon, because SYBR Green also detects any double-stranded DNA including primer dimers, contaminating DNA, and PCR products from misannealed primers. Contaminating DNA or primer dimers would show up as a peak separate from the desired amplicon peak. The expression ratio of each gene to *GAPDH *mRNA was calculated to adjust for variations in the RT-PCR reaction. All values are presented as means ± SEM. QPCR was replicated as follows: for the Day 45 and Day 95 samples, QPCR data were collected in biological duplicate from n = 3 animals and technical duplicates (n = 2) were performed on one animal sample (six data in total). For the Day 135 samples, QPCR data were collected in biological duplicate (n = 2) and technical duplicate (n = 2) from one animal sample (four data in total). Statistical analysis was performed using one-way ANOVA followed by the Tukey-Kramer multiple-comparison test. Differences were considered significant at *P *< 0.05.

**Table 3:** Oligonucleotide primers used for QPCR analysis

Gene	Primer	Sequence	Position
*oPRP1*	Forward	5' ATATGCCCAGGGCAAACTGT 3'	294–313
(AB231297)	Reverse	5' AATCGAAGGCATTGGTTTGG 3'	358-339
*oPRP2*	Forward	5' TGGAAGATGTACGAGGCTCGT 3'	590–610
(AB231298)	Reverse	5' CGCCTATCTTCATCGCTGGA 3'	631-612
*oGAPDH*	Forward	5' GCCATCACCATCTTCCAGGA 3'	81–100
(AF030943)	Reverse	5' CCACGTACTCAGCACCAGCA 3'	150-131

### Production and purification of recombinant proteins

The *oPRP2 *sequences encoding the mature-protein region, which combined the FLAG and 6 × His epitope tag sequences, were inserted into a pFLAG-CMV-3 vector (Sigma). The constructed plasmid was transfected into HEK 293 cells using FuGENE 6 (Roche Diagnostics, Basel, Switzerland) for transient transfection. Stably transfected HEK 293 cells were adapted to suspension culture in a spinner flask using 293 SFM II medium (Invitrogen, Gibco) and cultured in an atmosphere of 5% CO_2 _in air at 37°C for 3 days. The medium was separated by centrifugation.

Recombinant FLAG-tag and 6 × His-tag fusion proteins were purified using the 6 × His-tag portion. Approximately 1 liter of conditioned medium was processed at a time. Medium to which 1 ml Ni Sepharose 6 Fast Flow (Amersham Bioscience, Buckinghamshire, UK) was added was mixed and equilibrated with 20 mM sodium phosphate buffer, pH 8.0, containing 300 mM NaCl and 20 mM imidazole. Only the 6 × His-tag proteins bind to the Ni Sepharose 6 Fast Flow carrier. The medium with carrier was chromatographed on a PD-10 column (Amersham Bioscience). The fractions with carrier were washed with 20 mM imidazole. The fractions were eluted with 250 mM imidazole.

### Western blot analysis

One ng of purified protein was loaded on each lane, separated by SDS-PAGE, and electrophoretically transferred on to a polyvinylidene-difluoride membrane [58]. Western blotting was performed using the method of Towbin et al. [59]. Briefly, the membrane was blocked in 10% skimmed milk overnight and incubated with mouse anti-FLAG M2 (Sigma) for 1 h at room temperature, followed by incubation with anti-mouse IgG conjugated with alkaline phosphatase (Sigma) (diluted 1:3000) for 1 h at room temperature. Immunopositive bands were stained using NBT (Bio-Rad, Hercules, CA, USA) and BCIP (Bio-Rad).

### Bioassay of lactogenic activity using Nb2 lymphoma cells

Lactogenic activity was assessed by the rat Nb2 lymphoma cell proliferation assay [60,61]. Nb2 lymphoma cells were routinely grown in Dulbecco's modified Eagle's Medium (DMEM) and Ham's F12 combined medium (1:1) (Sigma) supplemented with 50 μM 2-mercaptoethanol, 100 U/ml penicillin and 100 μg/ml streptomycin, and containing both 10% HS and 10% FBS (maintenance medium: MM), in an atmosphere of 5% CO_2_/95% air at 37°C. Twenty-four hours before initiation of the assay the cells were harvested, washed with supplemented Fischer's medium containing only 10% HS (stationary medium: SM) and diluted to 1 × 10^6 ^cells/ml. At the initiation of the assay, cells were washed and aliquotted into 16-mm wells (1 × 10^6 ^cells/ml/well) of a 24-well culture plate. Ovine prolactin (oPRL: positive control), bPL (positive control), bPRP1 and oPRP2 preparations were added at various concentrations and the cells were incubated for an additional 72 h. Samples of treated cells were collected and counted in a Sysmex Microcell counter (Model CC-110; TOA Medical Electronics, Tokyo, Japan). Treatments were performed in quadruplicate.

## Authors' contributions

KU participated in the design of the study, carried out the mRNA cloning, QPCR and in situ hybridization studies, and wrote the manuscript. TT participated in the design and coordination of the study and performed the recombinant protein production, Western blotting and Nb2 bioassay. KU, TT and MH collected the sheep tissue samples. KO and MH carried out the preparations for natural mating and all animal care. KH participated in the design and coordination of the study and helped to draft the manuscript. All authors read and approved the final manuscript.
